# Supplementary material for: Linear breakwater reefs of the greater Caribbean: Classification, distribution & morphology
Source: PLoS One. 2022 Nov 23;17(11):e0270053. doi: 10.1371/journal.pone.0270053 (PMC9683561; doi:10.1371/journal.pone.0270053)
Supplement: S1 File — (DOCX) [file pone.0270053.s001.docx]

**Supporting Information**

**S1 File**

**Kmz files of all linear breakwater reefs (>500 m) in the Greater Caribbean**:

[Bahamas](https://drive.google.com/file/d/1WW3SN1XG77JTBoBQciAIbd6OSD9salJw/view?usp=sharing)

[Belize](https://drive.google.com/file/d/1DczgdUbVzlMCQPgzNaUMBWf2spLNndMP/view?usp=sharing)

[Central America](https://drive.google.com/file/d/1mpwJdWRltlCy7Ywk08iOpb2p0088iImh/view?usp=sharing)

[Colombia](https://drive.google.com/file/d/1539KRgf7yx-MmacZNvkcduXFNchl6D3V/view?usp=sharing)

[Cuba](https://drive.google.com/file/d/1O4JhaLXgSOxblzS9aixARBTMT5xRQwB6/view?usp=sharing)

[East Caribbean](https://drive.google.com/file/d/1tDQT18CHeXBQ-LmGoJJ4a2896D6FuyLf/view?usp=sharing)

[Florida](https://drive.google.com/file/d/1pc3xpzUhCuE9_vre-ttF-FdRoae3UqX0/view?usp=sharing)

[Hispaniola](https://drive.google.com/file/d/1UOG9-CC1CabgDGUpYDfgR96iZbnRsoyb/view?usp=sharing)

[Jamaica-Cayman](https://drive.google.com/file/d/1twh_w_OxeMfUwL-fNMAjbv32l4cJ_-ot/view?usp=sharing)

[Mexico](https://drive.google.com/file/d/1eK5vBuLZHe_50imdNdUw5DCCutgEc8QK/view?usp=sharing)

[Panama](https://drive.google.com/file/d/1Nnu_usScxEEyLObAoHJaX8ChVV1kQObh/view?usp=sharing)

[Puerto Rico](https://drive.google.com/file/d/1aWm6ZGAq2gK6f1vQAx6pZxAnA0Ox1Jbf/view?usp=sharing)

[South America](https://drive.google.com/file/d/1vn-e9UrKd6LT-goZ_JxJKXqbJ1HjYQjq/view?usp=sharing)

[Venezuela](https://drive.google.com/file/d/15mlNrIPHVleQHZyzCbAVIciNKDRMNty4/view?usp=sharing)

**Number and length (km) of linear breakwater reefs (>500 m) in the Greater Caribbean**, showing the longest unbroken reefs for each of the main reef types.

| **Total number** | 1,023 | **Total length** | 2,237 km |
| --- | --- | --- | --- |
|  |  | **Mean Length** | 2.2 km |
| **Fringing Reef**  **(Top 3)** | **1)** Belize, Lighthouse Reef: 33 km | **Faro**  **(top 3)** | **1,2,3)** Belize,  Rhomboid Shoals: 10 km  Rhomboid Shoals: 9 km  Rhomboid Shoals: 9 km |
|  | **2)** Mexico, Chinchorro Bank: 30 km |  |  |
|  | **3)** Bahamas, Great Inagua Island: 22 km |  |  |
| **Barrier Reef**  **(Top 3)** | **1)** Gulf of Mexico, Alacranes: 34 km | **Atoll**  **(top 2)** | **1)** Belize, Rhomboid Shoals: 23 km  **2)** Bahamas, Hogsty Reef: 16 km |
|  | **2)** Belize, South Cut to Gladden Spit: 27 km |  |  |
|  | **3)** Belize, Glovers Reef:  17 km |  |  |

**Cross validation test for independent classification levels.** Constrained canonical approach shows the strength of the association between the multivariate morphometric properties and the classification levels maximising separation of the two groups at the three levels of discriminant analysis for A: Setting, B: Type and C: Shallow Geomorphology in one dimensional ordination plots. Plots are ordered by decreasing percentages of consistency in morphometric parameters from A to D.

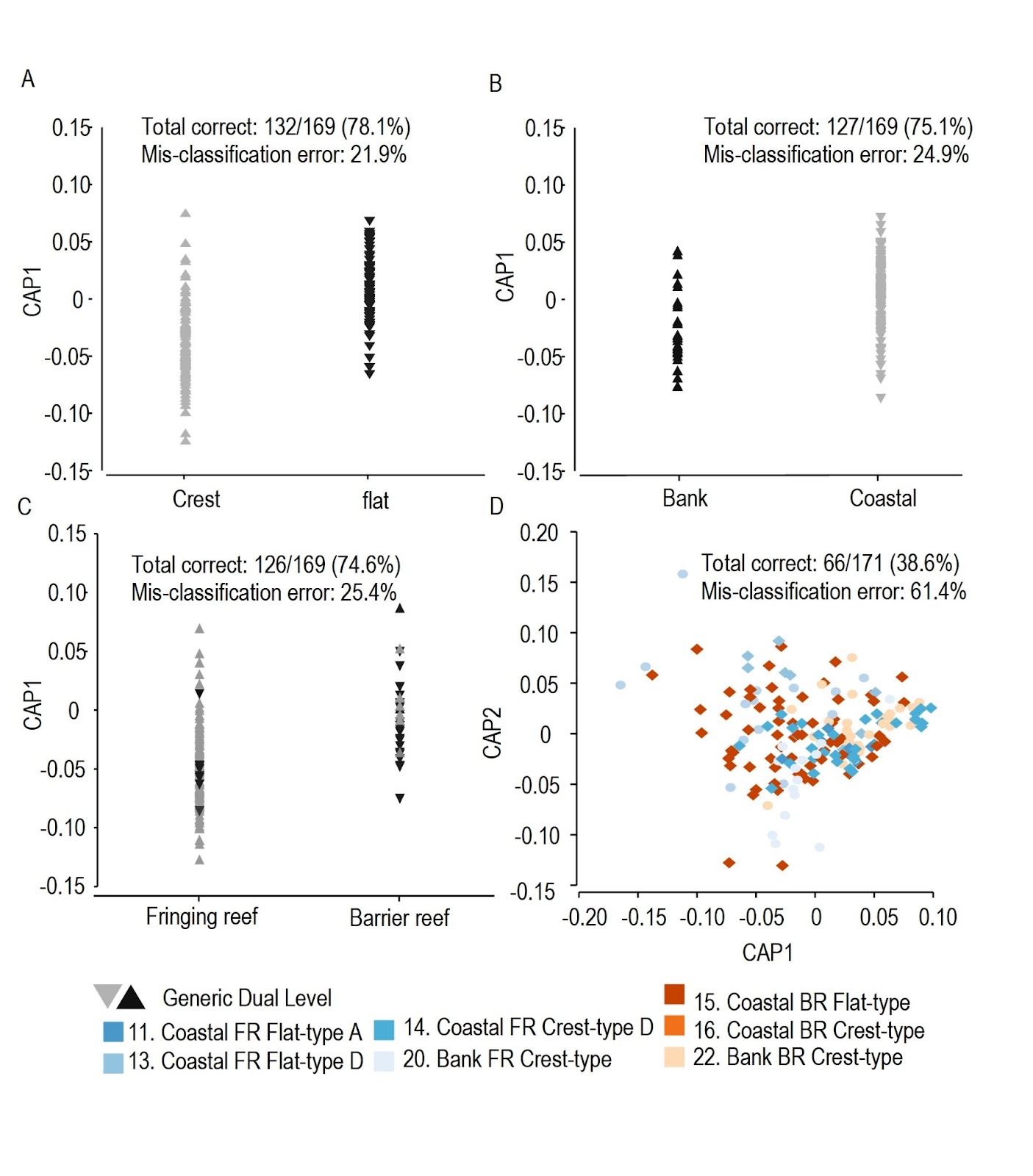


**Definition of reef zones**

The *crestline* (CL) is defined as the linear intertidal zone over which fairweather waves break. Its position is delineated by fairweather-wave breaking and substrate character in Google-Earth imagery. A contiguous intertidal CL will force wave breaking over a short distance, and waves will not cross it without significant modification. A discontinuous CL will break fairweather waves at specific points which are consistent between Google-Earth images, and gaps will allow wave passage and produce diffraction patterns in the lagoon. Larger swells and storm waves will break deeper on the reef-front than fairweather waves, making a CL look wider and more continuous, so care is needed in defining its persistence. Even under calm conditions, small non-breaking waves are perturbed as they pass over the CL, showing clear changes in wavelength and vector which can be used in Google-Earth imagery to help delineate CL position. This position can also be delineated in wave-free images by the breaker zone which is revealed by changes in the texture or tone of the benthic substrate (as on rocky-shores).

The *back-reef* (BR) is defined as the sand/gravel and/or coralgal substrate immediately leeward of the CL. Its lagoonal border is either delineated by a slope break (BR break) into the deeper lagoon and/or the onset of permanent seagrass meadows (Fig 2). Where seagrass and/or a clear slope break are absent, the transition from back-reef sand to a lagoonal substrate is used to delineate the BR limit, although this can be more subjective. The BR boundary can also be uncertain where intersected by lagoonal patch reefs or exposed to high-energy wave fields. We distinguish two geomorphic types of back-reef: a predominantly subtidal type which slopes from the CL to the lagoon, and an intertidal type which is predominantly sub-horizontal. A good proxy for distinguishing the depth of an intertidal BR is wave-transit character. Waves breaking on the CL will generate trains of standing waves over microtidal intertidal flats which propagate in orientations parallel to the CL and with modified vectors than the approaching breakers (Nakaza and Hino 1991). So the boundary between these two wave fields can help delineate both the CL and the intertidal depth of the BR zone. Deepening subtidal back-reef zones by contrast do not permit breakers to reform.

The *reef front* (RF) is defined as the contiguous coralgal substrate immediately seaward of the CL, extending downslope across the fore-reef shelf, and typically contains one or more subzones such as the spur-and-groove. The seaward boundary of the RF commonly forms a slope-break (RF Break) which is marked by minor sediment accumulation as shifting sand on the fore-reef shelf is trapped against the RF break (Fig. 2). In addition, changes in benthic substrates from the high-rugosity coralgal RF to the smoother bedrock of the fore-reef shelf can also define its limit. Areas where the RF is non-contiguous or patchy are not included in the RF area, as this indicates the absence of a well developed reef deposit (and the presence of coral grounds). In other areas, the RF can be extensive and cover the fore-reef shelf down to the mid-shelf break or even merging with submerged reefs along the shelf edge (cf. Blanchon and Jones 1995).

Additional Reference: Nakaza, E., & Hino, M. Bore-like surf beat in a reef zone caused by wave groups of incident short period waves. *Fluid Dynamics Research* **7,** 89-100 (1991).

**YouTube Video** showing common reef types:

https://youtu.be/dIr67dFwtO4
