# Supplementary material for: Linear breakwater reefs of the greater Caribbean: Classification, distribution & morphology
Source: PLoS One. 2022 Nov 23;17(11):e0270053. doi: 10.1371/journal.pone.0270053 (PMC9683561; doi:10.1371/journal.pone.0270053)
Supplement: Attachment — Submitted filename: Response to Reviewers PLOS.docx [file pone.0270053.s002.docx]

**Response To Reviewers**

**We thank the reviewers for their comments and pointing out typos.**

Reviewer #1: There is an adage that most models are either too good to be true or too true to be good. The former are so general that their descriptive and predictive powers are weak, whereas the latter are so intricate and detailed that each case is its own, ungeneralizable category. In this paper, Blanchon et al. provide a hierarchical classification of coral reefs of the Caribbean. Using Google Earth and bathymetric charts, they quantify the occurrence of the reef types by ecoregion and make a first cut at analyzing the morphometric variability of the different types they recognize. Their classification is far more detailed and rigorous than the simple fringing (no lagoon), bank/barrier (narrow lagoon), barrier (broad lagoon), and atoll (or carbonate-bank; enclosed lagoon) categories commonly in use by ecologists. Their contribution is potentially game-changing because it could dispense with a lot of less-precise classifications that have been published, adding rigor to the study of reef geomorphology. It also got me thinking about the nexus of reef geology and reef ecology, per the next paragraph.

Is this more detailed classification indeed more useful, or is it too true to be good? Clearly, this work will be of value to the authors and other reef geologists interested in understanding the physical controls on reef development. The two paragraphs starting on Line 541 amply demonstrate why this work is interesting and important, and they lay the groundwork for future tests of important geological hypotheses. But what about those pesky ecologists, who will likely have a hard time learning all the categories—and learning to use them effectively? The authors would serve themselves well to expand the Discussion a little, adding a few sentences to explain that it might be possible to make ecological predictions as consequences of the physical drivers of reef development. Alternatively, maybe the phenomena arrayed on ecological hierarchies transcend or are not affected by these differences of geomorphological classification (see for example Murdoch and Aronson, 1999, Coral Reefs). It would be fascinating to test the limits of the hypothesis that physics controls (almost) everything, as I suspect it does; but is the control geomorphological or the parochial result of environmental idiosyncrasy? These kinds of questions make the paper important.

Agreed. We have now included a short paragraph in the discussion covering the ecological implications of the crest and flat morphotypes

Specific Comments.

Comment 1: do not abbreviate sea level as SL, especially in the abstract. It is not a good look. DONE

Comment 2: Geister (1977) is cited, appropriately, but Hubbard’s 3-D environmental model of reef development is missing. That model might or might not be a counterpoint to what Blanchon and colleagues are trying to get across in their paper. Either way, it should be cited if only for completeness. It is certainly germane to the paragraphs of the Discussion beginning on lines 530 and 557.

Agreed. We have now included a short paragraph in the discussion covering the ecological implications of the crest and flat morphotypes. We have referenced the original source of Hubbards 3D model, which is Adey and Burke 1977.

Comment 3: the word data is sometimes used as a singular. Please go through the manuscript and correct that.

DONE

Line 125: allow, not allows. DONE

Line 164: faros, not faro’s DONE

Fig. 2: This figure consists of two panels, but the caption appears only to describe the top panel. A few words about the bottom panel would be appropriate, especially because the view is orthogonal to the view in the top panel. Where is the reef in the photo? DONE

Line 209: The definition of tract is at variance with its common use to describe the totality of reef development in southern Florida: the Florida reef tract. Is there an alternative word the authors can employ to avoid confusion?

Agreed. We removed this from the paper as it is an unnecessary descriptor.

Line 229: CL, BR, RF…this sentence sounds terrible when read aloud. Why not just spell out the terms to make it easy on the reader? That is what’s done in lines 231–232. DONE

Line 382: The sentence beginning with Whereas is not a sentence. Combine it with the previous sentence using a comma. DONE

Reviewer #2: A well-written and interesting paper with a logical initial approach to categorizing reef morphology using Google Earth that can be applied to regions outside the Caribbean. I find little to fault and have only three minor comments and list a few typo/formatting issues.

1. Strictly for reproducibility, if feasible indicate whether the bathymetry for each reef was sourced from the public domain or proxy.

Clarification: Individual reef data are provided as kmz files in the SI. We could annotate each of the 1023 reef files but feel it is laborious and unnecessary given that Admiralty charts are widely available for most areas in the Greater Caribbean.

2. Line 336, it would be useful to briefly summarize how the ecoregions were determined for those unfamiliar with Spalding et al.’s work – such as myself.

Clarification: Spalding et al’s 2007 work on reef ecoregions is widely cited and is an update from the 2001 UNESCO book World Atlas of Coral Reefs published in 2001. The boundaries used in both are arbitrary and our use of them is merely to divide the Greater Caribbean to facilitate comparison. Hence we think it serves no purpose to comment on how the boundaries were determined in the original paper since it bears no significance for our work.

3. Line 346, I am not sold on “15 Coastal BR flat-type” reefs dominating the Central East, East, Southeast and Southwest Caribbean ecoregions. From a purely visual analysis of your distribution using Figure 3, types 14 and 22 have a much higher dominance than 15, which appears to be present only in the Central East at a lesser extent than type 14. Including type 15 needs a better argument or explanation.

Agreed, we are only including 11 and 13 flat types as 15 has a much lower abundance and have altered the ms to reflect this.

• Line 149, check font size and line space formatting DONE

• Line 170-171, font color. I think maybe your list numbers were classified as references. DONE

• Line 204, should this be Supplementary Information SI-2? DONE

• Line 227, check line space formatting DONE

• Line 304, should this refer to Supplementary Table S1? DONE

• Line 471, should this be supplementary figure S1? DONE
